# An Update in Cystic Fibrosis-Related Diabetes in Children and Adolescents

**DOI:** 10.3390/children10121879

**Published:** 2023-11-30

**Authors:** Dana-Teodora Anton-Păduraru, Alina Mariela Murgu, Mădălina Andreea Donos, Felicia Trofin, Alice Nicoleta Azoicăi, Paula Popovici, Aurelian Bogdan Stana, Ionela Gheorghiescu, Laura Mihaela Trandafir

**Affiliations:** 1 Department of Mother and Child Medicine, “Grigore T. Popa” University of Medicine and Pharmacy, 700115 Iasi, Romania; dana.anton@umfiasi.ro (D.-T.A.-P.); alina.murgu@umfiasi.ro (A.M.M.); alice.azoicai@umfiasi.ro (A.N.A.); paula.popovici@umfiasi.ro (P.P.); aurelian.stana@umfiasi.ro (A.B.S.); laura.trandafir@umfiasi.ro (L.M.T.); 2 “Sf. Maria” Children Emergency Hospital, 700309 Iasi, Romania; 3 Department of Preventive Medicine and Interdisciplinarity—Microbiology, “Grigore T. Popa” University of Medicine and Pharmacy, 700115 Iasi, Romania; felicia.trofin@umfiasi.ro; 4 Clinical Hospital of Infectious Diseases “Sf. Parascheva”, 700116 Iasi, Romania; 5 Faculty of General Medicine, “Grigore T. Popa” University of Medicine and Pharmacy, 700115 Iasi, Romania; ionela.gheorghiescu@yahoo.com

**Keywords:** cystic fibrosis, complications, cystic fibrosis-related diabetes, children, adolescents

## Abstract

This paper delineates several aspects of cystic fibrosis-related diabetes (CFRD)—a common complication of cystic fibrosis (CF). CFRD exhibits a predilection for older individuals with CF, yet it also extends its influence on children and adolescents. Scientific insights postulate a potential link between CFRD and the aberrant mucus production within the pancreas, thereby culminating in pancreatic insufficiency. This, in turn, perturbs the synthesis of insulin, a pivotal endocrine hormone responsible for the regulation of glycemic levels. Standardized protocols advocate for the systematic screening of CFRD among all individuals with CF, commencing at the age of 10 years using the oral glucose tolerance test (OGTT). Therapeutic modalities encompass insulin therapy, dietary adjustments, and the vigilant monitoring of glycemic parameters. The overarching objective is to maintain blood glucose levels within a targeted range to mitigate the advent of diabetic complications. Untreated or sub-optimally managed CFRD can precipitate a spectrum of deleterious health ramifications, encompassing cardiovascular afflictions, neuropathy, renal dysfunction, and ocular complications.

## 1. Introduction

Diabetes represents an endocrine disorder, constituting a comprehensive classification and encompassing metabolic conditions characterized by the perturbation of glucose homeostasis. The principal organ overseeing the regulation of blood glucose levels is the pancreas. Over the long term, hyperglycemia engenders vascular complications, renal dysfunction, retinopathy, neuropathy, diminished life expectancy, comatose states, or even mortality. The most prevalent categories of diabetes are type 1 (T1DM) and type 2 (T2DM), with a nascent acknowledgement of diabetes of the exocrine pancreas (DEP), also known as pancreatogenic diabetes or type 3c diabetes, which materializes consequent to afflictions of the exocrine pancreas, such as acute or chronic pancreatitis, alcoholic pancreatitis, or pancreatic cancer. This type also fits cystic fibrosis-related diabetes (CFRD).

The motivation for the present review article regarding CFRD in pediatric and adolescent populations is manifold and serves to achieve several crucial objectives within the academic and clinical sphere. Firstly, it seeks to engage in knowledge synthesis by collating and summarizing the extant research and insights concerning CFRD as it manifests in children and adolescents. This imperative arises due to the intricate and dynamic nature of CFRD, necessitating a comprehensive compendium of the contemporary state of knowledge to benefit healthcare providers, researchers, and caregivers.

Secondly, the article serves as a source of clinical guidance, particularly for healthcare professionals such as pediatricians, endocrinologists, and pulmonologists. It strives to furnish them with updated information, specialized to the needs of children and adolescents with cystic fibrosis who may develop diabetes, encompassing the aspects of screening, diagnosis, management, and treatment. Furthermore, it underscores the importance of early detection and intervention, recognizing the profound impact CFRD can have on the health and quality of life of young patients with cystic fibrosis. This focus on early diagnosis and the adherence to pertinent clinical guidelines is indispensable in optimizing patient outcomes. Through a comprehensive review of the existing literature, the article seeks to elucidate the research gaps and future directions, thereby guiding prospective research endeavors and clinical investigations. This aids in the continuous advancement of our comprehension and therapeutic approaches to this condition. Additionally, the article embraces the role of education and awareness dissemination, with the intent of elevating awareness among healthcare practitioners, patients, and their families regarding the unique challenges and considerations associated with CFRD in the younger demographic. Enhanced understanding by all stakeholders is pivotal for promoting more effective disease management and improved patient outcomes.

By catering to the informational needs of the parents and caregivers of children afflicted by both cystic fibrosis and CFRD, the article seeks to support caregivers and families. It aims to provide clear and accessible information that empowers them to better comprehend the challenges posed by these conditions and the potential interventions available for their children. Policy and guideline development represents another crucial function of the review article. It offers valuable insights that can inform the formulation of clinical guidelines, treatment protocols, and healthcare policies tailored specifically to CFRD in children and adolescents. This is pivotal for ensuring that patients receive optimal care and that healthcare systems are equipped to cater to the distinctive requirements of this demographic. Lastly, the review article serves as a vital resource for advocacy and funding endeavors, supporting advocacy groups and organizations in their pursuit of financial support and resources for research and therapeutic advancements in the realm of CFRD in children and adolescents.

## 2. Materials and Methods

**Figure 1:**
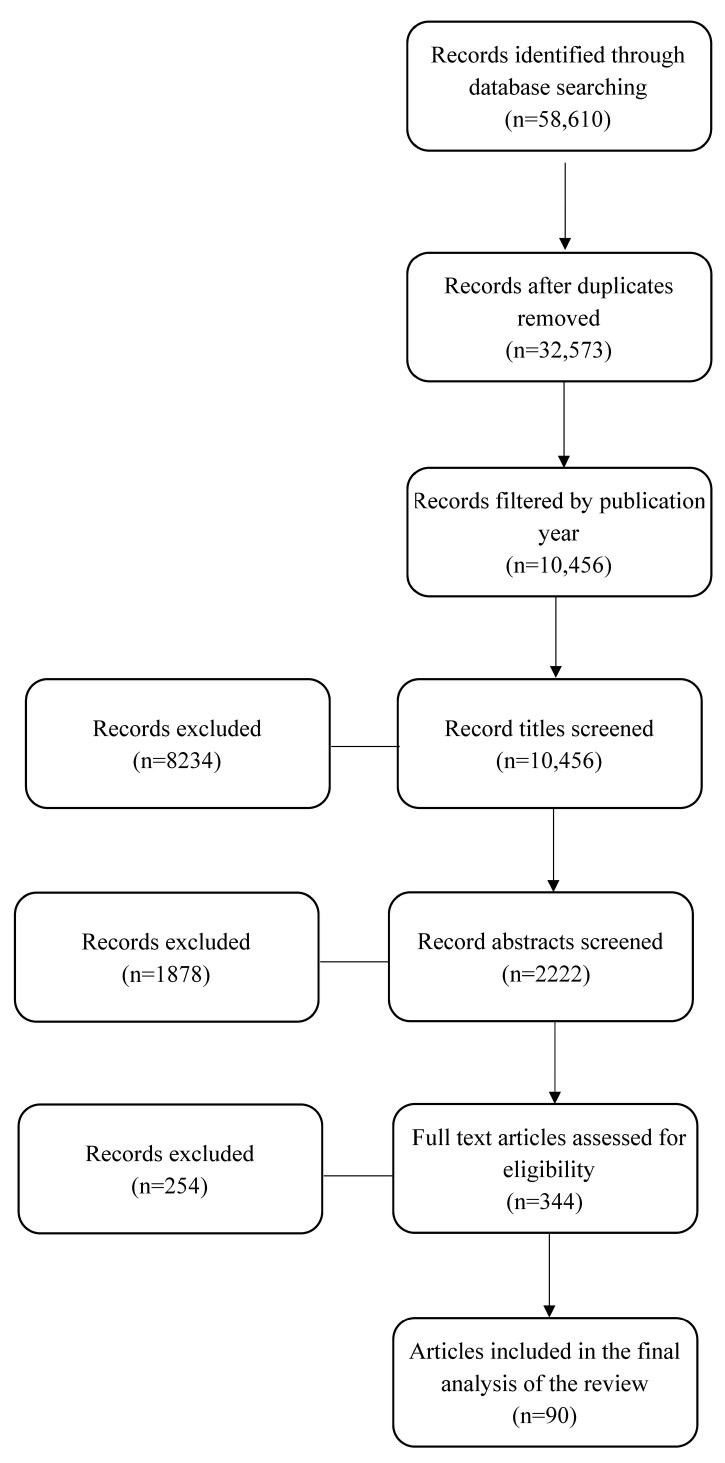
The review flowchart.

This comprehensive review encompassed a total of 90 studies. Initially, relevant articles were identified using a search of the database with the keywords “cystic fibrosis-related diabetes”, “children”, and “adolescents”. Subsequently, 55,978 articles were excluded following a meticulous screening of duplicates, publication years, and titles as they did not align with the research objectives outlined in this paper. Upon conducting an in-depth examination of the abstracts, additional articles were excluded from consideration based on various factors, including their relevance to the topic, accessibility constraints, or their overall pertinence to the research inquiry. Following this stage, a thorough review of the complete texts led to the exclusion of 1878 articles due to considerations such as their relevance, methodological incongruity, misalignment with the scope of the study, issues related to quality and credibility, or language barriers. The accompanying flowchart (Figure 1) visually illustrates the sequential progression of information throughout the different stages that was inherent to our review process.

The procedure for the identification and curation of the articles for inclusion in our review adhered to a stringent set of criteria. These criteria encompassed an alignment with our central research question, namely, “Is cystic fibrosis-related diabetes a complication of cystic fibrosis? What is new?”. This was in addition to an adherence to our research objectives, consideration of the publication year, categorization of scientific research, and an evaluation of the quality in the presentation of the findings. Systematic searches were conducted utilizing established databases, including PubMed and Google Scholar, between June 2023 and October 2023. The selected studies underwent a qualitative analysis that scrutinized their adherence to the principles of scholarly composition, clarity, brevity, citation frequency, sample size, the provision of pertinent data, the articulation of results, and the formulation of conclusions. These individual facets were amalgamated and incorporated into the final narrative synthesis.

## 3. Definition

CFRD represents a heterogeneous condition that shares certain attributes with both T1DM and T2DM, notably involving anomalies in insulin secretion and peripheral insulin resistance. However, CFRD constitutes a distinct clinical entity [1]. This differentiation is grounded in its unique physiopathology, which must be understood in clinical attributes, thereby necessitating a specialized approach in terms of its systematic screening, diagnosis, and management, as exemplified in Table 1 [2,3,4].

## 4. Epidemiology

The prevalence of CFRD exhibits an age-dependent pattern and displays variability among the studies, with a positive correlation observed between the prevalence and screening rates, as noted by Ballmann in 2021 and Olesen et al. in 2020 [5,6]. While CFRD is uncommon before the age of 10, early abnormalities in glucose metabolism become apparent from a young age, warranting the need for screening at an earlier stage, as emphasized by Olesen et al. [5]. The prevalence of AGT is notably higher in children between the ages of two and four compared to those over five years old. This observation suggests that disruptions in carbohydrate metabolism can manifest quite early in the lives of these patients, with the severity of glucose homeostasis dysregulation potentially exhibiting age-dependent characteristics [1]. Several investigations have detected early glucose tolerance irregularities in individuals with CF, employing parameters such as 1-h glucose values from oral glucose tolerance tests (OGTTs) and control glucose monitoring (CGM). These findings have been corroborated by Brodsky et al. in 2011, Chan et al. in 2018, and Elidottir et al. in 2021 [7,8,9]. In line with these observations, Granados et al. (2019) asserted that children with an impaired glucose tolerance (IGT) are at a heightened risk for the early onset of CFRD [2].

While the prevalence of CFRD in children stands at a modest 2%, it escalates with age, with approximately 20% of adolescents and 30% to 50% of adult CF patients developing CFRD, as reported by Kayani et al. in 2018 and Chan et al. in 2021 [10,11]. This prevalence surges to nearly 80% among patients older than 40, particularly those harboring severe genotypes, according to findings by Moheet et al. in 2022 [12]. A European study conducted in 2020 yielded a prevalence of 0.8% in patients under the age of 10 and 9.7% in the 10 to 19-year-old group, as highlighted by Olesen et al. (2020) and Mogoi et al. (2022) [5,13]. In line with these trends, the most recent annual report from the European CF Society (ECFS) registries divulged a CFRD prevalence of 22.2% across Europe. This figure represents a significant increase compared to the 2015 prevalence data, which reported a rate of 0.8% in patients under 10 years, 9.7% in those aged 10–19, 24.1% in the 20–29 age group, and 32.7% in individuals over 30 years [5,14]. Hadjiliadis et al. (2005) observed a notable increase in CFRD prevalence from 28.6% before transplant to 50% after transplant, a phenomenon potentially attributed to the physiological stress induced by surgery, post-transplant infections, and medications [15].

Concerning CFTR modulator therapy, its impact on CFRD prevalence is relatively low. For instance, Volkova N et al. (2020) found that the prevalence of CFRD among patients treated with Ivacaftor (IVA) stood at 12.1% in the United States and 2.4% in the United Kingdom when compared to the group not receiving IVA, where the prevalence was 18.3% in the United States and 8.2% in the United Kingdom [16].

The risk factors contributing to the onset of CFRD encompass exocrine pancreatic insufficiency (PI), female gender, the degree of residual cystic fibrosis transmembrane regulator (CFTR) function, severe CFTR genotypes (Class I, II, and III mutations), genetic modifiers (SLC26A9, PRSS1), a family history of T2DM associated with a threefold increased risk, CF-related liver disease (CFLD), solid organ transplantation, systemic corticosteroids use, calcineurin inhibitors (tacrolimus and cyclosporin), deteriorating lung function, a history of allergic bronchopulmonary aspergillosis (ABPA), gastrostomy tube feedings, and lower childhood height z-scores [5,12,17,18,19,20,21]. Notably, certain genes implicated in T2DM (TCF7L2) are also associated with CFRD, and both conditions are characterized by the accumulation of amyloid particles in beta cells, which is indicative of cellular stress, a phenomenon underscored by Blackman et al. in 2013 and Granados et al. in 2019 [2,22].

## 5. Pathophysiology of CFRD

The pathophysiology of CFRD is intricate, not fully elucidated, multifaceted, and characterized by heterogeneity, yet it diverges from T1DM. Notably, the autoimmune pathogenesis typically associated with DM1 is not a hallmark of CFRD, as indicated by Moran et al. in 2010 [19].

Numerous studies have been conducted to elucidate whether the fundamental defect in the CFTR protein exerts an influence on the functionality of pancreatic beta cells, yielding a divergent body of evidence. The outcomes have, thus far, presented contradictions regarding whether the manipulation of the CFTR channel function, either through its blockade or enhancement, imparts any discernible impact on insulin secretion [2,23]. Alternative investigations have illuminated the indirect repercussions of CFTR dysfunction on beta cells, leading to insulinopenia, and direct repercussions on alpha cells, contributing to the development of glucose intolerance in the context of CF. Conversely, some research posits that the CFTR protein assumed a role in the secretion of both insulin and glucagon, in addition to safeguarding beta cells from oxidative stress [10,24].

Mutations within the CFTR gene have been associated with an increased susceptibility of beta cells to oxidative stress. Moreover, CFTR gene mutations exert an influence on glucagon secretion from pancreatic alpha cells, manifesting as reduced glucagon levels and an inadequate response to arginine stimulation or hypoglycemia. This alteration in the negative feedback mechanism governing glucagon release further contributes to the gradual establishment of glucose intolerance over time [2,4]. Notably, the presence of decreased immunoreactive trypsinogen levels in individuals with severe CFTR genotypes is associated with an elevated risk of developing CFRD [10].

The CFTR genotype holds the potential to predispose individuals to diabetes through various mechanisms. One pathway involves a direct impact attributed to exocrine pancreatic dysfunction, while an alternative perspective posits that the CFTR defect itself could be the primary cause of impaired glucose metabolism. The CFTR gene is intricately involved in the development of pancreatic parenchyma and exerts an intrinsic influence on both alpha and beta cells. Dysfunctional CFTR channels render beta cells more vulnerable to oxidative stress, a condition that can subsequently lead to apoptosis and the initiation of diabetes [4].

The emergence of CFRD is attributed to multiple factors, encompassing a reduction in the islet cell mass and β-cell dysfunction leading to deficiencies in insulin and glucagon. The principal etiology of CFRD primarily arises from insulin deficiency, which occurs as a consequence of islet damage. The morphological studies conducted on pancreatic tissues obtained from neonates, infants, and young children with CF revealed that, among those under four years of age, the relative beta cell areas and the percentage of beta cells were reduced to approximately 50%. However, it is noteworthy that the incidence of diabetes and its severity did not exhibit a direct correlation with the proportion of residual beta cells. Among the other causal factors for CFRD, fibrosis and fatty infiltration within the pancreatic tissue play a significant role, characterized by the phenomenon known as “beta cell strangulation” [25]. In this context, the islets of Langerhans, which remain functional, gradually become isolated due to the development of fibrous bands, leading to distortions in the blood supply to these areas. Notably, the extent of fibrosis within the pancreas did not exhibit a direct correlation with the severity of diabetes, indicating that the other factors influenced the function of the remaining functional beta cells [2]. Fat maldigestion in CF patients contributes to the pathophysiology of CFRD and is further exacerbated by liver and gallbladder pathology [10].

In individuals with CF, the initial phase of insulin secretion, which is dependent on depolarization, experiences a delay, resulting in postprandial hyperglycemia. This, in turn, leads to impaired glucose tolerance and eventually culminates in the development of CFRD. F508del homozygotes tend to display reduced peak insulin secretion in the OGTT, a delay in the first phase of insulin secretion in the intravenous glucose tolerance test, and decreased tissue sensitivity to insulin in comparison to F508del heterozygotes [4]. Over time, the glucose tolerance progressively deteriorates, transitioning from indeterminate glycemia into impaired glucose tolerance, CFRD without fasting hyperglycemia, and eventually CFRD with fasting hyperglycemia [2]. Notably, the second phase of insulin secretion, which is independent of depolarization, remains functionally intact [10,12,26,27]. These deficiencies may sometimes be exacerbated by insulin resistance, a feature associated with the chronic inflammatory pulmonary state prevalent in these patients.

Inflammation also plays a pivotal role in the pathophysiology of CFRD. Recent investigations have unveiled the presence of several inflammation mediators within pancreatic islet cells. In both T2DM and CFRD, elevated concentrations of inflammatory markers were noted, and systemic inflammation was associated with the development of tissue insulin resistance [1]. One such mediator, IL1-beta, known for its involvement in beta cell apoptosis in both T1DM and T2DM, has also been identified in pancreatic cells affected by CF, potentially rendering it a valuable therapeutic target. Additional mediators of inflammation encompass interleukin 6 (IL6), CXCL10, TNF-alpha, and IFN-gamma [2]. The elevated levels of circulating inflammatory mediators have been associated with impaired glucose tolerance in CF patients. The persistence of inflammation in these individuals fosters tissue damage, contributing to widespread organ dysfunction and failure. This state of inflammation leads to pancreatic damage, the immune infiltration of other islet components, and insulin resistance, ultimately resulting in an altered glucose tolerance and the development of CFRD. Consequently, CFRD appears to be a consequence of beta cell loss, accompanied by inflammation within the islets [6,25]. Notably, Hart et al. (2018) reported intense peri-islet and intra-islet immune infiltration within human CF pancreases and islets [28].

Analogous to T2DM, CFRD exhibits peripheral insulin resistance in cells, coupled with intracellular amyloid deposition. Hepatic insulin resistance and accelerated gluconeogenesis contribute to heightened hepatic glucose production, culminating in a hyperglycemic state [1]. Patients with CFRD often experience peripheral insulin resistance due to reduced glucose uptake by muscle tissue and hepatic insulin resistance, characterized by the inability to effectively suppress hepatic glucose production. Insulin resistance may be further exacerbated by factors such as acute pulmonary exacerbations, severe chronic lung disease, and the administration of systemic glucocorticoid therapy [29].

Another endocrine system implicated in the onset of CFRD, with a consequential impairment of beta cell function, is the incretin axis. The incretin hormones, namely the glucose-dependent insulinotropic peptide (GIP) and glucagon-like peptide 1 (GLP-1), are intestinal hormones that are released into circulation following food intake, particularly meals that are rich in carbohydrates. These hormones serve a multitude of biological functions, including stimulating insulin release into the bloodstream, inhibiting glucagon and somatostatin production, safeguarding the mass of pancreatic beta cells, retarding gastric emptying, and suppressing appetite. Some research studies have posited that individuals with CFRD exhibit significantly reduced levels of GIP and GLP-1 when compared to their healthy counterparts. This diminishment likely contributes to a heightened postprandial glycemic variability, given the concurrent impairment of the initial phase of insulin secretion in diabetes [2]. Incretin secretion and the responsiveness to incretins are notably compromised in cases of pancreatic insufficiency (PI) stemming from CF [30]. The gradual decline in the pancreatic exocrine function precipitates damage to the pancreatic islets, ultimately culminating in the emergence of CFRD [10].

**Figure 2:**
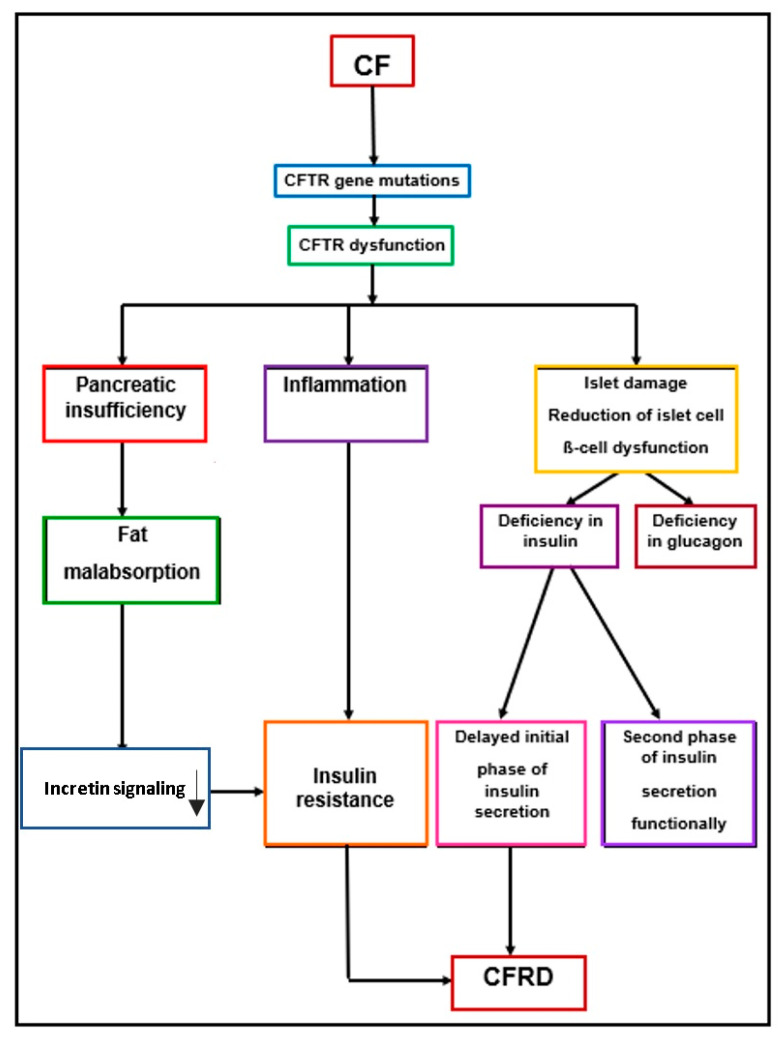
Pathophysiology of CFRD. Abbreviation: ↓ = deacreased.

Figure 2 briefly shows the pathophysiology of CFRD.

## 6. Clinical Considerations

CFRD—a distinctive variant of diabetes, is characterized by a subtle and gradual onset that may be heralded by an extended period of progressive insulin inadequacy [11,12]. CFRD frequently presents with an absence of overt symptoms, leading to prolonged periods of undiagnosed status. As highlighted by Schiaffini et al. (2023), various forms of overt diabetes and prediabetes can exhibit a convergence of early glucose metabolism anomalies [31]. Acute and abrupt onsets, typified by ketoacidosis, are exceedingly rare, although they may be encountered in certain instances, such as during respiratory infections or when corticosteroids therapy is administered, accentuating the tissue resistance to insulin. Notably, several classical symptoms of diabetes, including polyuria and polydipsia, may partially overlap with the characteristic symptoms of CF. The increased water consumption observed in many CF patients is often attributed to dry mouth. The additional manifestations of CFRD encompass sensations of fatigue, involuntary weight loss, or an inability to sustain an ideal body weight despite a high caloric intake, growth retardation, and a delayed onset of puberty [4].

The commencement of CFRD is intricately linked to the progression of the underlying CF condition, often manifesting several years before an official diagnosis is made. The existing literature highlights a decline in the respiratory function and nutritional status of CF patients commencing approximately six years before the CFRD diagnosis. The study conducted by Perrem et al. in 2019 demonstrated an association between CFRD and adverse outcomes, such as diminished lung function, negative effects on the nutritional status leading to a reduced body mass index (BMI), and a history of allergic bronchopulmonary aspergillosis (ABPA) [32].

Initially, hyperglycemia may manifest during pulmonary exacerbations or periods of corticosteroid therapy. As the condition advances, asymptomatic postprandial hyperglycemia becomes apparent, primarily detectable through OGTTs [2,4]. In cases where individuals exhibit hyperglycemia that arises during an acute illness, is verified through laboratory plasma glucose measurements, and persists for several weeks, it is advisable to conduct a routine screening approximately six weeks after recovery. The onset of diabetes is deemed to have occurred when patients first meet the diagnostic criteria [4].

## 7. Screening and Diagnosis

Early glucose irregularities are a prevalent occurrence, particularly among adolescents. Consequently, individuals presenting early glucose abnormalities should undergo routine screening [4]. The criteria employed for the diagnosis of prediabetes and CFRD have been adapted from those utilized in the detection of early microvascular complications in T2DM. However, these criteria may not be entirely suitable for identifying clinical deterioration in CFRD [4].

### 7.1. Oral Glucose Tolerance Test (OGTT)

Currently, the established standard for screening and diagnosing CFRD is the OGTT. Both the Cystic Fibrosis Foundation (CFF) and the American Diabetes Association (ADA) recommend conducting this test annually for all CF patients aged 10 years and older [10,31,33]. Although the International Society for Pediatric and Adolescent Diabetes (ISPAD) guidelines recommend initiating CFRD screening at the age of 10 years, some studies propose an earlier start, either from the age of 6 years or after 3 years following the CF diagnosis. This is supported by data indicating that even at the age of 10, 55% of children already presented early glucose abnormalities [34]. High-risk patients with a 1-h glucose level exceeding 200 mg/dL (11.1 mg/dL) are advised to undergo more frequent screenings every six months [34].

The OGTT should be performed following a minimum 8-h fasting period, during which patients ingest a standard glucose solution at a dosage of 1.75 g/kg dextrose (up to a maximum of 75 g) within a 5-min interval [35]. The CFF recommends a 2-h OGTT, involving glucose level measurements at time 0 (fasting) and 120 minutes post-ingestion of the glucose-containing beverage.

According to criteria established by the ADA, ISPAD guidelines, and international guidelines for the screening and management of CFRD in children and adolescents, the OGTT results can be categorized as follows.

- Normal glucose tolerance (NGT): Characterized by fasting plasma glucose levels (FPG) of <5.6 mmol/L (<100 mg/dL) and plasma glucose at 2 h of <7.8 mmol/L (<140 mg/dL).
- Indeterminate glucose tolerance (INDET): Manifests when both the fasting and 2-h plasma glucose levels are normal, but the 1-h plasma glucose value is ≥11.1 mmol/L (≥200 mg/dL).
- Impaired fasting glucose (IFG): Indicates fasting glucose levels between 5.6–7 mmol/L (100–126 mg/dL) with 2-h plasma glucose remaining normal.
- Impaired glucose tolerance (IGT): Characterized by fasting glucose levels between 5.6–7 mmol/L (100–126 mg/dL) and 2-h plasma glucose levels between 7.8–11.0 mmol/L (140–199 mg/dL).
- CFRD without fasting hyperglycemia: Features fasting glucose levels of <7 mmol/L (<126 mg/dL) and 2-h plasma glucose levels ≥ 11.1 mmol/L (≥200 mg/dL).
- CFRD with fasting hyperglycemia: Noted when the fasting glucose levels are >7 mmol/L (>126 mg/dL) and the 2-h plasma glucose levels are ≥11.1 mmol/L (≥200 mg/dL) [4,14,31,34,36,37].

The occurrence of hypoglycemia (defined as a glucose level under 50 mg/dL) during an OGTT in CF patients has been documented in 15% of cases [38]. Hypoglycemia in CF patients might be linked to factors such as delayed initial insulin secretion, liver disease, malnutrition, gastrointestinal complications, and dysfunctions related to the incretin hormones [29]. In the context of adolescent CF patients, a 1-h glucose value equal to or exceeding 140 mg/dL is considered an early indicator of CFRD risk, while the 2-h glucose level is predictive of lung function deterioration [34].

The OGTT is the preferred method of screening for CFRD. However, it is associated with several drawbacks, which are as follows.

- Time-consuming process: The OGTT demands a significant amount of time for both patients and medical staff.
- Fasting requirement: The patients must fast before undergoing the test, which can be burdensome.
- Morning testing: It is typically conducted in the morning, restricting the flexibility of timing.
- Taste and tolerance issues: Some patients may find the taste of the glucose solution unpalatable or may have difficulties tolerating it.
- Multiple blood draws: The procedure involves a substantial number of punctures or blood sample collections.
- Poor adherence: Adherence to the OGTT is low in many CF centers [2,35].

The CFF Patient Registry report highlighted a suboptimal patient compliance with OGTT screening. In 2017, only 61.7% of CF patients aged 10 to 17 years, who were not previously diagnosed with CFRD, underwent annual diabetes screening via an OGTT. The poor adherence to the OGTT can be attributed to multifactorial determinants, necessitating the exploration of alternative, more user-friendly screening methods [39].

### 7.2. Fasting Plasma Glucose (FPG)

Although the FPG levels are employed in the diagnosis of DM, they are not suitable for identifying CFRD. In 1997, the ADA acknowledged FPG levels equal to or greater than 7 mmol/L (or 126 mg/dL) as one of the diagnostic criteria for CFRD. Nevertheless, relying solely on FPG measurements for CFRD screening remains unreliable because FPG concentrations can persist within the normal range for an extended duration in approximately half of CFRD patients [14,21].

For CFRD diagnosis, the established criteria include FPG levels at or above 7.0 mmol/L (or 126 mg/dL), random plasma glucose concentrations equal to or exceeding 11.1 mmol/L (or 200 mg/dL), and an HbA1c level of 6.5% or higher [14]. In the context of CFRD diagnosis, it is important to distinguish between transient hyperglycemia that occurs at the onset of a disease exacerbation, which rapidly resolves with appropriate treatment, and persistent hyperglycemia. If hyperglycemia persists for more than 48 h despite appropriate therapy, it warrants a CFRD diagnosis. For some CF patients who require enteral nutrition to maintain an adequate body weight, CFRD may be suspected when the blood glucose surpasses 11.1 mmol/L (200 mg/dL) during or after enteral nutrition on two separate days [2].

### 7.3. Glycated Hemoglobin (HbA1c)

In 2010, the ADA incorporated HbA1c as a potential tool for screening and diagnosing CFRD. Nevertheless, HbA1c, similar to FPG, is ill-suited for CFRD diagnosis, as it exhibits a sensitivity of merely 50% and does not correlate adequately with the mean plasma glucose levels. In the context of CFRD, transient postprandial hyperglycemia does not significantly impact the red blood cell glycation status, which can lead to low HbA1c levels. This may be due to increased red blood cell turnover influenced by inflammation that is often present in individuals with cystic fibrosis [10,21]. Consequently, HbA1c exhibits a limited predictive value, and not all CFRD cases are identified when relying on HbA1c measurements [14]. In comparison to T1DM, the target HbA1c value for CFRD should be lower, as mean plasma glucose levels do not exhibit a strong correlation with HbA1c [6].

Diagnosing CFRD during an OGTT may yield disparate results when compared to the low values of HbA1c. This divergence can be attributed to the fundamental disparity between the OGTT and HbA1c levels, which represent distinct aspects of glucose intolerance. These markers are not interchangeable [40]. Unlike T2DM, where an HbA1c value greater than 6.5% serves as a diagnostic criterion, approximately 70% of patients with confirmed CFRD through an OGTT exhibit normal HbA1c values [41]. Consequently, HbA1c measurements cannot act as a substitute for an OGTT in CF patients. Following the establishment of a CFRD diagnosis, HbA1c is a valuable tool for monitoring glycemic control. However, the increasing adoption of CGM techniques has been observed for this purpose [4,6].

### 7.4. Other Screening Methods

Fractional serum fructosamine (FSF) and glycated albumin (GA) represent the glycated proteins that offer alternative markers for evaluating glycemic control in patients with diabetes. Importantly, these markers are not influenced by the lifespan of red blood cells, making them valuable tools for assessing diabetes-related metabolic functions [21]. FSF, which displays a positive correlation with the OGTT results and a negative association with FEV1, has proved to be an effective tool for screening CFRD. Particularly in situations where HbA1c measurements may be unreliable due to red blood cell turnover, FSF has emerged as a reliable alternative for tracking clinical outcomes [42].

GA, on the other hand, is formed through the non-enzymatic glycation of plasma albumin and occurs at a rate ten times faster than hemoglobin glycosylation. This characteristic positions GA as a useful marker for identifying postprandial hyperglycemia and pronounced glycemic fluctuations. It is nine times more efficient than HbA1c and is unaffected by an accelerated red blood cell turnover. Moreover, GA offers the advantage of representing glycemic control over the past 14–21 days. It is important to note that GA can be influenced by certain disorders, such as protein abnormalities, uric acid, urea, and other low-molecular-weight substances, as well as the albumin duration. Notably, GA has shown to be associated with the development of diabetes complications like retinopathy and nephropathy, rendering it a potential marker for assessing atherosclerosis risk and coronary artery diseases [43].

Another alternative method for assessing glycemic control is 1,5-anhydroglucitol (1,5-AG), a naturally occurring dietary polyol. Its reduction can reflect both overall and postprandial hyperglycemia, providing insights into short-term glycemic control over two weeks [21,39]. Tommerdahl et al. (2019) concluded that biomarkers, such as HbA1c, 1.5AG, and GA, had limited effectiveness in identifying CFPD and CFRD when compared to the OGTT. As for HbA1c, they suggested that a lower threshold might be necessary to enhance its sensitivity and specificity relative to GA [39].

Chan et al. (2018) suggested that the HbA1c performance was consistent in CF, which was comparable to that in healthy individuals as well as in adults with T1DM or T2DM. Moreover, the other biomarkers also demonstrated correlations with the mean blood glucose values, as recorded by CGM, with GA exhibiting a similar effectiveness to HbA1c [8]. Nevertheless, it should be noted that in small-scale studies, none of these markers displayed a concordance with the OGTT results or HbA1c levels. Therefore, they are not recommended for screening or diagnosing CFRD, possibly leading to an oversight of early glucose abnormalities [21].

### 7.5. Continuous Glucose Monitoring (CGM)

The utilization of CGM is gaining increased recognition and promotion, heralding a significant transformation in the screening and management of CFRD [44]. CGM proves to be a valuable instrument for illuminating disturbances in glucose homeostasis and establishing a CFRD diagnosis. It enables a precise quantification of glucose peaks and troughs, as well as the proportion of the time glucose levels exceed the predefined thresholds. Originally designed for monitoring the efficacy of insulin pump therapy in individuals with T1DM, CGM has been validated for detecting early glucose tolerance abnormalities in patients with CF [14,29].

Despite the growing interest in employing CGM for the early detection of glucose abnormalities in CF patients, no studies have yet lent support to its use as a screening or diagnostic tool for CFRD [21]. Notably, CGM detects hypoglycemic events more frequently compared to the OGTT, and hyperglycemia detected using CGM appears to correlate with the intermediate glucose elevations observed during the OGTT [6,21].

In comparison to the OGTT, CGM offers the advantage of providing a more detailed characterization of glucose patterns in free-living conditions throughout one to two weeks [11]. This approach allows for the examination of glycemic control within real-life settings, as it derives data from the interstitial glucose levels recorded by a sensor placed on the patient’s arm [43]. Nevertheless, it is essential to note that the glucose levels measured in the interstitial fluid may deviate from the blood glucose values and are unsuitable for diabetes diagnosis [9].

It has been observed that anomalies in glucose homeostasis identified by CGM are correlated with declining lung function, pulmonary inflammation, a higher incidence of *Pseudomonas aeruginosa* infection, weight loss, and unfavorable health outcomes among CF patients. Additionally, these anomalies predict a worsening of glucose tolerance along with increasing insulin deficiency [45,46,47,48]. Elevated glucose values exceeding 200 mg/dL, as detected by CGM, have been shown to predict the future development of CFRD [46].

Regarding the interpretation of the recorded values, most of the reference values are derived from those established for T1DM and T2DM. Official guidelines for the use of CGM in the context of CFRD have yet to be defined. For instance, in patients with T1DM and T2DM, the guidelines recommend maintaining blood glucose levels between 70–180 mg/dL for 60–70% of the duration of the recordings. However, for the individuals with CFRD, the target blood glucose values should aim to approximate the ranges of normality, albeit requiring individualized adjustments on a case-by-case basis [44]. Table 2 shows the results of some studies regarding the methodologies for screening and diagnosing CFRD.

## 8. Complications Development

The remarkable advancements achieved in the management of CF over the last six decades have notably elevated the life expectancy of the afflicted individuals, with some attaining the age of 50, particularly in developed nations. Nonetheless, prolonged life expectancies and enhanced diabetes screening engender inadvertent ramifications, leading to complications that may augment the mortality rates. CFRD, recognized as the most prevalent extrapulmonary ailment accompanying CF, is associated with elevated morbidity and mortality among CF patients. Left untreated, CFRD can lead to a decline in lung function and reduced survival rates [30].

Patients diagnosed with CFRD encounter challenges in maintaining their body weight and pulmonary function, as documented by Kayani et al. in 2018 [10]. The onset of CFRD is accompanied by an exacerbation of the deterioration of the nutritional status and pulmonary functions attributed to insulin deficiency and recurrent lung disease exacerbations, resulting in an increased number of hospitalizations, a higher prevalence of infections from *P. aeruginosa* and *Burkholderia cepacia*, and diminished overall physical health. Moreover, several studies have demonstrated that clinical deteriorations can initiate up to 4–5 years before a formal diagnosis of diabetes is made, as indicated by Patel et al. in 2021 [58]. CFRD is associated with reduced life expectancy and elevated mortality and morbidity rates, with no observable gender-based disparities in mortality, according to the findings of Ballmann (2021) and Suppakitjanusant et al. (2023) [6,59]. The nutritional status and body weight are pivotal factors for forecasting mortality, with a favorable nutritional status linked to more positive outcomes, as underscored by Kayani et al. in 2018 [10]. A value of HbA1c ≥ 6.5% corresponds to a threefold increased risk of death, as documented by Ballmann (2021) [6]. Nevertheless, in recent years, with increased OGTT screening, earlier diagnoses, and comprehensive treatments, a decrease in diabetes-related mortality has been observed, per the findings of Schwarzenberg et al. in 2007 [60]. The timely detection and the optimization of glycemic management hold the potential to enhance the nutritional status, diminish the incidence of respiratory exacerbations, and extend the lifespan.

The mechanisms through which CFRD detrimentally influences prognosis entail a combination of protein catabolism, reduced lean body mass, and malnutrition stemming from insulin inadequacy, along with an elevated pro-inflammatory and pro-infectious state correlated with intermittent hyperglycemia, as proposed by Sandouk et al. in 2021 [61]. Thus, it is imperative to maintain an adequate nutritional status for preserving lung function and ensuring long-term survival. A gradual decline in the BMI is also a hallmark of diabetes, but early screening and insulin therapy appear to ameliorate the clinical course [2]. The decline in the respiratory function commences 2–4 years before the diabetes diagnosis and is attributed to augmented protein catabolism due to insulin deficiency. In a healthy state, glucose should not be detectable in respiratory secretions. However, in children with CFRD, an increase in blood sugar above 144 mg/dL is linked to the appearance of glucose in respiratory mucus, a phenomenon that fosters the growth of pathogens like *P. aeruginosa*, the predominant pulmonary pathogen in CF patients, thereby promoting pulmonary exacerbations. Alterations in the blood glucose levels contribute to oxidative stress and inflammation, which can lead to microvascular dysfunction in the lungs, according to Totani et al. in 2017 and Tomlinson et al. in 2022 [62,63]. Brodsky et al. in 2011 identified that for every 10 mg/dL increase in blood glucose one hour after commencing the OGTT forced expiratory volume in one second (FEV1) decreased by 1% [7].

While CFRD shares similarities with T1DM and T2DM concerning the risk of microvascular complications, it is notably distinct in that macrovascular complications are rarely observed. The primary cause of mortality among CFRD patients is generally attributed to pulmonary complications related to the underlying CF pathology, with a scarcity of reported cases related to atherosclerotic disease [2].

Microvascular complications, such as diabetic retinopathy, nephropathy, and neuropathy, can manifest in CFRD. However, these complications tend to be less frequent and severe in comparison to other types of diabetes, primarily due to the persistent presence of minimal endogenous insulin secretion. Therefore, regular screening for such complications is deemed essential [2]. Notably, microvascular complications in CFRD tend to develop later, typically after a minimum of 10 years, following the onset of CF disease progression [6,10].

Autonomic neuropathy is a prevalent condition, affecting approximately 52% of CFRD patients. This condition leads to a reduction in cardiorespiratory reflexes and has a direct impact on the phrenic nerve and inspiratory muscle innervation, subsequently resulting in decreased lung function. This was documented by Ticona et al. in 2023 [24]. A study by Andersen et al., cited by van den Berg et al. in 2023, found that peripheral neuropathy was observed in only three transplanted patients [64].

In the context of CFRD, diabetic retinopathy has been documented in 5–27% of CF patients and typically becomes evident after approximately 12 years of CF disease progression [24]. Additionally, Schwarzenberg et al. reported in 2007 that 16% of CFRD patients displayed retinopathy. Furthermore, the same study reported that the percentage of CFRD patients with hypertension was lower in adults, while those with a CFRD duration exceeding 10 years, fasting hyperglycemia, and microalbuminuria accounted for 14%, and the percentage of individuals with nephropathy was greater in comparison to T1DM and T2DM [60]. The use of intravenous aminoglycosides for treating respiratory exacerbations and prolonged immunosuppressive therapy after lung transplantation are potential contributors to renal damage in the long term. Microalbuminuria is associated with an increased risk of chronic kidney disease. It is essential to interpret microalbuminuria cautiously since it can be falsely elevated in cases involving antibiotics, severe illness, or physical stress, according to Kempegowda et al. in 2020 [65].

Atherosclerotic disease is infrequently observed in CFRD, possibly owing to the “cardio protective” effects of CF characterized by a lean body habitus and low cholesterol levels [61]. However, some CFRD patients exhibit increased arterial stiffness, which, in conjunction with hyperlipidemia, hypertension, and other external risk factors, may contribute to the development of cardiovascular disease [10,24]. The current guidelines advocate for an annual screening for nephropathy, retinopathy, and neuropathy, commencing 5 years after the CFRD diagnosis. Alternatively, if the exact onset of diabetes is unclear, screening should begin upon the detection of fasting hyperglycemia [2,60].

Macrovascular complications are rarely reported in CFRD. Although hypertriglyceridemia is increasingly common, cholesterol levels tend to remain low. Elevated triglycerides are associated with a heightened insulin resistance and glucose intolerance. Regular lipid profile assessments are advisable for CFRD patients, particularly those with a personal history of obesity or a family history of coronary heart disease. Routine evaluations should encompass blood pressure measurements, with optimal values ranging from 130 mmHg for systolic blood pressure to 80 mmHg for diastolic blood pressure or lower than the 90th percentile for age and sex. Arterial hypertension is a significant risk factor for diabetic nephropathy [2].

## 9. Treatment of CFRD

The management of CFRD necessitates a personalized approach tailored to the unique clinical presentation and severity of each case. This personalized care should be orchestrated through the collaborative efforts of a multidisciplinary healthcare team. The overarching goal of CFRD treatment is to mitigate the airway glucose levels and counteract the decline in the pulmonary function and nutritional status [10].

The multifaceted treatment strategy for CFRD comprises several key components, including insulin therapy, nutritional support, physical activity, and comprehensive patient education on blood glucose self-monitoring, as well as education and family involvement. These factors are integral to therapeutic success and should be complemented by the continuation of CF-specific treatments [66].

### 9.1. Insulin Therapy

The primary and recommended treatment modality for children and adolescents diagnosed with CFRD is insulin. It is noteworthy that children with CFRD exhibit a higher sensitivity to insulin compared to those with T1DM [31]. Insulin, an anabolic hormone, plays a critical role in regulating glucose metabolism. Its inadequate secretion not only results in hyperglycemia but also leads to the depletion of muscle mass and protein reserves. Consequently, insulin therapy is the optimal approach for managing CFRD [67]. Clinical studies have shown that insulin therapy has the potential to reverse clinical deteriorations, improve respiratory function, and enhance BMI, underscoring the importance of initiating insulin therapy at an early stage [30,68]. However, it is essential to acknowledge that the heterogeneity of CF as a clinical entity means that not all cases of CFRD require exogenous insulin [3].

Presently, several clinically effective insulin regimens are available, and the choice of regimen should be tailored based on the patient’s glycemic fluctuations, disease severity, and the specific insulin preparations at hand, in accordance with insights from Ode et al. in 2019 [67]. These diverse regimens include long-acting insulin administered once daily, intensified insulin treatment involving multiple daily injections with prandial insulin supplements, typically using rapid-acting insulin analogs, or insulin pump therapy that delivers continuous basal insulin with boluses at mealtimes [31,69]. It is important to note that a consensus regarding the optimal insulin regimen for CFRD management has yet to be established [6]. Customizing the treatment regimen should consider the degree of hyperglycemia and the timing of the hyperglycemic events (i.e., fasting or postprandial).

In comparison to children with T1DM, children with CFRD typically exhibit lower daily insulin requirements. The initiation of insulin therapy for CFRD often commences with a basal insulin dose of 0.2 IU per kilogram per day, and/or a prandial insulin dose ranging from 0.05 to 0.1 IU per kilogram before each principal meal [31]. All insulin regimens necessitate a precise evaluation for both carbohydrate intake and the patient’s clinical condition to make the necessary dose adjustments.

For individuals with CFRD experiencing hyperglycemia á jeun, the therapeutic regimen necessitates the use of rapid-acting insulins, such as aspart, lispro, or glulisine, administered approximately 15–30 min before meals. Meanwhile, fasting periods are managed with long-acting insulins, such as glargine, degludec, or detemir. To optimize the nutritional status and effectively regulate the glycemic fluctuations, insulin is recommended with every meal and snack. A starting insulin dose of one unit per 30 g of carbohydrates can be considered, which is in line with Ode et al. in 2019 [67]. In the case of CFRD patients without fasting hyperglycemia, it has been observed that using rapid-acting insulin solely before meals may help prevent weight loss. Consequently, this regimen is endorsed by the current guidelines [67].

These treatment protocols do come with certain drawbacks. One significant limitation is the requirement for a substantial number of blood samples to monitor blood glucose levels, which may impact the patient’s adherence to the treatment [67]. Another drawback is the risk of hypoglycemia, with its prevalence in CFRD comparable to that in T1DM [10]. Exercise can lead to reduced insulin requirements, while factors like growth, development, pulmonary exacerbations, and exacerbations of the underlying disease may necessitate increased insulin doses [67].

An alternative to intensive multi-injection insulin therapy is the utilization of insulin pumps, which have a demonstrated efficacy in achieving glycometabolic control [31]. In this approach, fast-acting insulin is exclusively employed. This method provides is adjustable for snacks, nocturnal feeds, and carbohydrate content of meals [30]. The insulin pump operates by subcutaneously infusing insulin at a predetermined basal rate, typically measured in units per hour. Another advantage is its ability to modify the basal dose as needed throughout the day. The insulin-to-carbohydrate ratio is calculated similarly to the previously mentioned regimen. Blood glucose monitoring is still recommended before and after meals, along with before bedtime. Furthermore, the integration of CGM techniques can be employed in conjunction with insulin pumps [67].

### 9.2. Nutritional Treatment

The nutritional management of CFRD is an integral aspect of the treatment, although it is insufficient as a stand-alone intervention without appropriate pharmacological therapy. In the case of children and adolescents with CFRD, dietary recommendations encourage a hypercaloric and hyperproteic diet, without imposing restrictions on carbohydrate, fat, or salt consumption, which is in line with the dietary guidelines for CF patients without diabetes. These dietary instructions diverge from those typically advised for individuals with T1DM [6,30,70].

Given the gastrointestinal complications frequently experienced by individuals with CF, a dietary regimen comprising three meals and three snacks daily is recommended. The daily caloric intake should range from 120% to 200% of that of the general population, with adjustments made to suit individual nutritional needs [10]. This higher caloric intake is crucial for maintaining an optimal nutritional status, ensuring adequate weight gain in the context of malabsorption, addressing the elevated energy expenditure, and normalizing the blood glucose levels [37,70].

Carbohydrates should contribute to approximately 45–50% of the daily energy intake, and they do not need to be restricted, which in contrast to the recommendations for T1DM or T2DM. The consumption of refined sugars and carbonated sweet beverages should be limited, as they can induce challenging-to-control hyperglycemia, even with the use of a rapid-acting insulin treatment. The intake of non-caloric sweeteners should also be moderated, as they do not provide calories and may hinder nutrient absorption [70].

Lipids, which account for 40% of the total daily caloric intake, are an essential component of the CFRD dietary regimen. The recommendation of a high-fat diet serves to counteract the challenges related to poor lipid absorption [10]. Notably, no instances of atherosclerotic disease have been documented among CF patients, justifying the absence of fat intake restrictions. Foods rich in omega-3 fatty acids are also encouraged to help address the omega-3 to omega-6 fatty acid imbalance and potentially influence inflammatory markers, although the supporting evidence is not yet conclusive. Lipids exert an indirect influence on blood glucose regulation by delaying gastric emptying and carbohydrate absorption, thereby delaying the peak of postprandial hyperglycemia. Patients are advised to steer clear of ketogenic diets, which may lead to unintended weight loss, as they rely on an exceptionally high fat intake that induces rapid satiety and may result in complications arising from lipid malabsorption [70].

Protein absorption can be comparably affected as lipid absorption. Despite the use of pancreatic enzyme replacement therapy, which only partially rectifies malabsorption, the protein requirements may need to be increased by up to 200% in comparison to those of healthy children and adolescents. This heightened protein intake is warranted due to the presence of an insulin deficiency, which leads to an elevated catabolic state characterized by an increased degradation of lipids and proteins. Unlike other forms of diabetes, protein intake is not restricted by diabetic nephropathy, and, in general, proteins have a limited impact on glycemic control [70].

Recognizing that CF patients experience increased salt losses through sweat during specific conditions (e.g., high temperatures, physical exertion, febrile states), there are no limitations placed on salt intake. Neglecting salt supplementation can result in hyponatremic and hypochloremic dehydration [37]. In light of the elevated losses of fat-soluble vitamins experienced by CF patients, it is commonly advisable to supplement their diets with fat-soluble multivitamins, including Vitamins A, D, E, and K.

### 9.3. Oral Hypoglycemic Agents

#### 9.3.1. Repaglinide

Repaglinide, a non-sulfonylurea hypoglycemic agent and a meglitinide analogue, exerts its action by blocking ATP-dependent potassium channels, which leads to an increased glucose-dependent insulin release from beta cells. This mechanism of action is also associated with a mild inhibition of CFTR chloride channels, as described by Ode et al. in 2019 [67]. The efficacy and safety of repaglinide for the treatment of CFRD in patients over the age of 10 were assessed in a multicenter, open-label, randomized trial reported by Ballmann et al. in 2018. The study findings concluded that repaglinide exhibited an effectiveness comparable to insulin, as evidenced by changes in the BMI, HbA1c, and pulmonary functions [71]. Moreover, the drug was well tolerated, making it a viable option for CFRD patients who may be averse to insulin therapy [6]. However, it is worth noting that a study by Kayani et al. in 2018 suggested that repaglinide, while having insulinogenic effects and reducing the postprandial glucose levels, was somewhat less effective when compared to insulin [10].

#### 9.3.2. Incretins

In the realm of antidiabetic medications belonging to the incretin mimetics class, these drugs act by mimicking the action of incretins, which are gut peptides that are secreted in response to nutrient intake. They stimulate beta cells, promoting insulin secretion in response to postprandial hyperglycemia [10].

Semaglutide, an antidiabetic drug from the incretin mimetics class, has been used in combination with basal insulin. At a low dose, it has demonstrated the potential to replace prandial insulin and effectively regulate glycemic levels. Another member of the incretin mimetics class, Sitagliptin, has proven to be a valuable option in the treatment of early stage dysglycemia and CFRD. It is well tolerated and does not carry the risk of hypoglycemia, as noted by Sebastian-Valles et al. in 2023 [56].

GLP-1 (glucagon-like peptide-1) agonists represent another category of antidiabetic agents. In CFRD, postprandial hyperglycemia and the absence of the first phase of insulin secretion are prominent issues. This aligns with a deficiency in the incretin effect, suggesting that therapies based on enhancing incretin action might be beneficial. GLP-1, considered to be the most clinically relevant incretin, stimulates the insulin release from pancreatic beta cells, inhibits glucagon secretion, and enhances gastric emptying. It is important to note that GLP-1 levels are relatively low in CF, though the available data are limited. A small systematic study explored the utility of GLP-1 agonists in CFRD, revealing an improvement in glycemic excursions, which were primarily attributed to delayed gastric emptying. However, it should be acknowledged that the use of GLP-1 agonists can be associated with gastrointestinal side effects such as diarrhea, bloating, and vomiting, which may limit their application in CF patients [10,67]. Importantly, various studies have not found an increased risk of pancreatitis or pancreatic cancer associated with treatment using GLP-1 receptor agonists in diabetic patients [72,73].

#### 9.3.3. Dipeptidyl Peptidase-4 (DPP-4) Inhibitors

DPP-4 inhibitors are considered a pragmatic alternative, given the gastrointestinal concerns linked to the use of GLP-1 agonists. DPP-4 inhibitors serve the purpose of preventing the swift inactivation of GLP-1, subsequently amplifying the secretion of endogenous insulin and curtailing glucagon secretion. However, despite numerous investigations, substantial empirical evidence substantiating the advantages of this category of antihyperglycemic agents remains elusive. Notably, several studies in this regard have been prematurely discontinued due to the challenges related to participant recruitment [67].

#### 9.3.4. Metformin

Due to the potential peril of lactic acidosis and the paucity of substantiated data regarding the clinical efficacy of metformin—the sole accessible biguanide, its prescription is discouraged for the management of CFRD [67].

### 9.4. Physical Activity

Engaging in physical activity is a pivotal component in the management of various diabetes types, including CFRD, as it serves to mitigate postprandial glycemic fluctuations as well as the overall daily glycemic levels, as stipulated by Ode et al. in 2019 [67]. In individuals undergoing insulin treatment, physical exercise may contribute to further reductions in blood glucose levels. The guidelines advise the consumption of 15–30 g of carbohydrates for each hour of physical activity. In the context of countering hypoglycemia, the “Rule of 15” is a recommended approach, involving the ingestion of 15 g of rapid-acting carbohydrates, followed by a blood glucose reassessment after 15 min. If the blood glucose levels remain low, a subsequent administration of the same carbohydrate amount is advised. Employing carbohydrate measurements can also be advantageous for patients with prediabetes or reactive hypoglycemia. Although the total carbohydrate intake is not diminished, it is suggested that carbohydrates should be distributed evenly throughout the day [70].

## 10. The Role of CFTR Modulators in Patients with CFRD

The CFTR modulators encompass the pharmaceutical agents designed to ameliorate and potentially reinstate the expression and function of the malfunctioning CFTR protein. These modulators enhance the CFTR channel conductance, exemplified by Ivacaftor (IVA), and rectify the protein’s trafficking to the cell surface, as observed in Lumacaftor (LUM), Tezacaftor (TEZ), and Elexacaftor (ELX). Their application has demonstrated noteworthy enhancements in lung functionality and a reduction in CF exacerbations. However, the precise implications of CFTR modulators on the extrapulmonary aspects of CF, notably glucose tolerance and insulin secretion, are currently a subject of debate in the scientific literature [74].

The precise mechanism by which CFTR modulator therapy influences the pathophysiology of CFRD remains incompletely understood. Their impact on CFRD appears to be an indirect consequence, primarily by restoring the CFTR function, thereby diminishing inflammation and augmenting the functionality of the pancreatic islets and insulin sensitivity. Notably, an increase in incretin secretion from gastrointestinal neuroendocrine cells may lead to improved insulin secretion. Furthermore, the potential enhancement of the caloric intake and intestinal absorption through CFTR modulator therapy may lead to weight gain, consequently contributing to elevated insulin resistance [75].

Despite the in vitro findings indicating that the CFTR modulators do not significantly affect human insulin secretion, certain evidence suggested a decrease in insulin secretion in the cells isolated from human and ferret pancreases, along with the cells derived from CFTR-deficient species. This reduction in insulin secretion might be associated with the limited specificity of the CFTR modulators, as revealed in the studies conducted by Putman et al. in 2023 [30].

The data published by Schmid et al. in 2014 suggested a potential preventive role for CFTR modulators in CFRD among CF patients. This study highlighted improvements in patients with glucose intolerance (IGT) and those exhibiting undiagnosed abnormal glucose indicators (INDET), both of which were considered prognostic indicators for CFRD [76].

### 10.1. The Role of Ivacaftor in CFRD

Ivacaftor, a modulator that is effective in patients with CFTR gating mutations, such as G551D, has shown substantial enhancements in the peak expiratory volume/second (FEV1), reduced exacerbation frequencies, improved nutritional status, and elevated quality of life. Notably, studies have also indicated the favorable effects of IVA on blood glucose in patients with these mutations. For homozygous patients bearing the F508del mutation, the combination of IVA with LUM or TEZ has been authorized. Nevertheless, the pulmonary and glycemic improvements were less pronounced in this context [55]. Research conducted by Regard et al. in 2023 further delineated the benefits of IVA in CFRD management, revealing an improved insulin secretion and positive effects on CFRD remission [77]. Prior investigations have demonstrated the effectiveness of IVA modulators in reducing blood glucose levels, occasionally even inducing CFRD remission in patients with specific CFTR mutations, particularly class III mutations [78].

### 10.2. The Role of Lumacaftor/Ivacaftor in CFRD

Investigations into the efficacy of Lumacaftor/Ivacaftor (LUM/IVA) dual modulators for patients with the F508del mutation have yielded mixed results, with some studies indicating a limited effectiveness [74,79]. A study involving 40 CF patients aged 12 years and older carrying the F508del mutation, who initially exhibited an impaired glucose tolerance or diabetes, reported improvements in glucose tolerance as evidenced by reductions in the 1-h and 2-h glucose levels during the OGTT after one year of LUM/IVA treatment. Additionally, a significant percentage of individuals witnessed a regression of CFRD following LUM/IVA therapy, as observed by Misgault et al. (2020) and Salazar-Barragan et al. (2023) [78,80]. However, these results were not replicated in either the US PROSPECT study examining LUM/IVA treatment, or a smaller study conducted in Italy, which included six patients with glycemic dysfunction or CFRD [77]. Similarly, other studies did not show favorable effects of this combination of modulators (Table 3). Merjaneh et al. (2022), specifically focusing on LUM/IVA therapy in F508del homozygous patients, did not indicate significant enhancements in CFRD or an impaired glucose tolerance [79].

### 10.3. The Role of Elexacaftor/Tezacaftor/Ivacaftor (ELX/TEZ/IVA) on CFRD

The combination of ELX/TEZ/IVA is recognized as a highly effective modulatory therapy for eligible CF patients. This combined therapy has gained approval for use in nearly 90% of the CF population aged six years and older. However, relevant studies are scarce in the existing literature concerning the evaluation of ELX/TEZ/IVA’s impact on CFRD and glucose metabolism. It remains unclear whether this modulatory therapy effectively treats glycemic dysfunction [75,77,78,81]. In an observational study conducted by Scully et al. (2022), improvements were observed in the blood glucose values and blood glucose variability using CGM after the initiation of ELX/TEZ/IVA therapy [46]. The modulation of the CFTR protein using the ELX/TEZ/IVA combination may positively influence the membrane transport of glucose transporter 4 (GLUT4) and ameliorate insulin resistance [78,81].

Salazar-Barragan et al. (2023), following a review of 13 studies that included both children and adolescents, as well as adults, concluded that the ELX/TEZ/IVA combination had beneficial effects on blood glucose compared to LUM/IVA, but with some variations among the individuals (some patients decreased their insulin dosage, others had incidents and hypoglycemia) [78]. In comparison to Lumacaftor/Ivacaftor (LUM/IVA) therapy, ELX/TEZ/IVA appears to exhibit more consistent and promising effects on the blood glucose levels, especially in patients with the most common CF mutation in the CFTR gene, F508del (Table 3) [82,83,84,85].

**Table 3:** The role of CFTR modulators in children with CFRD.

Study	Year	Authors	Study Type	Study Group Size (Patients)	Age (Years)	Modulator	Results	Reference
Effect of IVA on insulin secretion	2013	Bellin et al.	Pilot study	5	6–52	Ivacaftor	Improvement of insulin secretion	[83]
The effects of Ivacaftor on growth and pancreatic function	2019	Emery et al.	Retrospective	28	2–17	Ivacaftor	Mild decrease seen in random blood glucose, but without significance	[84]
Disease progression in patients treated with IVA	2020	Volkova et al.	Observational, prospective, multicenter	635		Ivacaftor	Lung function better preserves lower frequencies of exacerbations and hospitalizations; improved nutritional status	[16]
Effect of LUM/ IVA on glucose in CF	2019	Li et al.	Prospective	9	11–15.6	Lumacaftor/ Ivacaftor	Worsening in HbA1c and fasting plasma glucose; no significant improvements in the HbA1c levels, OGTT results, or CGM data after 29 weeks of treatment	[86]
Effect of LUM/IVA on glucose abnormalities in CF	2020	Misgault et al.	Observational, prospective, multicenter	40	24 ± 10	Lumacaftor/ Ivacaftor	Improvement of abnormalities in glucose tolerance after 1 year of LUM/IVA treatment	[80]
Effect of LUM/IVA on the insulin secretion in CF	2021	Moheet et al.	Prospective	39	22 ± 10	Lumacaftor/ Ivacaftor	No changes in fasting glucose, 2-h glucose, insulin, or time to peak insulin after 3, 6, and 12 months of treatment. Did not lead to improvements in insulin secretion or glucose tolerance	[87]
Effect of modulators on glucose in CF	2022	Korten et al.	Observational	16	≥12	Elexacaftor, Tezacaftor, Ivacaftor	Improvement of glucose tolerance measured by the OGTT	[81]
Impact of CFTR modulators on glucose metabolism.	2022	Piona et al.	Prospective, observational	21	≥6	Elexacaftor, Tezacaftor, Ivacaftor or Lumacaftor/ Ivacaftor	CFTR modulators do not significantly ameliorate glucose homeostasis and/or any of its direct determinants; insulin sensitivity worsened in the group treated with LUM/IVA	[88]
The effect of elexacaftor-tezacaftor-ivacaftor (ETI) on glucose tolerance	2022	Choudhari et al.	Retrospective	12	≥12	Elexacaftor, Tezacaftor, Ivacaftor	HbA1c values suffered minimally changes without significant differences comparative with the control group formed by children with CF who do not receive CFTR modulators	[89]
Effects of CFTR modulators on the FEV1, weight, BMI, HbA1c, and daily insulin dose	2023	Lurquin et al.	Retrospective	17	37 ± 12	Elexacaftor, Tezacaftor, Ivacaftor	Decrease in insulin doses; positive effects on BMI and FEV1	[23]
Glucose regulation after the initiation of CFTR modulator treatment	2023	Park et al.	Case series/ presentation	7	>12	Elexacaftor, Tezacaftor, Ivacaftor	Positive impact on glycemic control and insulin requirements	[90]

## 11. Research Gaps and Future Directions

Despite the advancements in the screening, diagnosis, and treatment of CFRD, persistent challenges necessitate attention in future studies. Notably, considering the suboptimal adherence to the OGTT as a screening method, the identification of the most efficacious screening and diagnostic approach for CFRD warrants dedicated investigation, particularly with the increasing prevalence of CGM in the CF population. Furthermore, given that individuals with CFRD maintain the same hypercaloric, high-protein, normoglucidic diet as those with CF without diabetes, it becomes imperative to assess the potential impact of this dietary regimen on the risk of CFRD, especially among overweight and obese individuals. In the realm of CFRD treatment, limited studies exist on the use of GLP-1 receptor agonists and DPP-4 inhibitors in children and adolescents. Consequently, investigations into the efficacy of these treatment modalities in CFRD are deemed necessary.

Prospective inquiries should also delve into the preventative aspects of initiating CFTR modulators at an earlier stage to ascertain if such interventions can forestall or delay the onset of CFRD. Multicenter studies in pediatric populations, including younger age groups, are crucial to comprehensively assess the clinical, biological, and developmental effects of various CFTR modulators. Moreover, the specific effects of these modulators on glucose metabolism and the pathogenesis of CFRD represent notable knowledge gaps that warrant further exploration. While the short-term effects of CFTR modulators are becoming better understood, their long-term implications on both pulmonary and extrapulmonary outcomes necessitate evaluation.

Given the prolonged life expectancy resulting from novel CFTR modulator therapies, an increased incidence of complications, including psychological issues such as depression and anxiety, is anticipated. Consequently, the implementation of educational programs targeting patients, families, and the multidisciplinary healthcare team managing this disease is recommended to address these emerging challenges.

## 12. Conclusions

With the increasing life expectancy of individuals affected by CF, attributed to advancements in screening initiatives and medical management, there has been a notable rise in the prevalence of comorbid conditions, notably CFRD—a significant risk factor that considerably augments morbidity and mortality in this population.

Due to the precocious presence of the abnormalities of glucose metabolism, it is necessary to explore the benefits of different therapies at the earliest ages to determine their role in the prevention or delaying of CFRD.

The judicious management of CFRD in the pediatric and adolescent cohort assumes a pivotal role in the preservation of their holistic well-being, and the incorporation of diabetologists within the multidisciplinary care team for CF patients, encompassing screening, monitoring, and treatment, is imperative.

## Figures and Tables

**Table 1:** Comparison between CFRD, T1DM, and T2DM (modified after Iafusco et al., 2021 [4]).

	CFRD	T1DM	T2DM
Prevalence in CF patients	22.2% (ECFS registries)	0.2%	11%
Maximum age of onset	Young adults	Children and adolescents	Adults
Onset	- Subtle and gradual - Frequent absence of overt symptoms	Acute	Insidious
Risk factors	- Exocrine pancreatic insufficiency (PI) - Female gender - Degree of residual CFTR function - Severe CFTR genotype - Genetic modifiers - Family history of T2DM - CF-related liver disease (CFLD) - Solid organ transplantation - Systemic corticosteroids use - Calcineurin inhibitors - Deteriorating lung function - History of allergic bronchopulmonary aspergillosis (ABPA) - Gastrostomy tube feedings - Lower childhood height z- score	- Family history of T1DM - Infectious agents - Intestinal microbiota - Dietary factors - Genetics - Age - Geography	- Family history of T2DM - Overweight - Prediabetes - Age > 45 years - Gestational diabetes - African American, Hispanic or Latino, or American Indian
Clinical symptoms	- Polyuria - Polydipsia - Sensations of fatigue - Delayed onset of puberty	- Polyuria - Polydipsia - Polyphagia - Loss of weight	- Increased thirst and hunger - Frequent urination - Unintended weight loss - Fatigue - Frequent infections - Acanthosis nigricans
Weight	Normal/Underweight	Normal	Overweight/Obese
Antibodies	Rare	Yes	No
Pathogenesis	- Inflammation, insulin resistance - Islet damage - Reduction in islet cells - β-cells dysfunction	- Autoimmune destruction of the β-cells of the endocrine pancreas	- Insulin resistance and reduced secretion of insulin by the β-cells
Insulin secretion	Reduced	Reduced to absent	Severely reduced
Sensitivity to insulin	Somewhat reduced	Somewhat reduced	Severely reduced
Screening/diagnosis	Oral glucose tolerance test (OGTT)	FPG ≥ 126 mg/dL (7.0 mmol/L), or a 2-h plasma glucose level ≥ 200 mg/dL (11.1 mmol/L) during a 1.75 g/kg oral glucose tolerance test (OGTT), or a random plasma glucose ≥ 200 mg/dL (11.1 mmol/L) in a patient with classic symptoms of hyperglycemia or hyperglycemic crisis	HbA1c
Risk of ketoacidosis	Rare	Yes	Rare
Insulin	Yes	Yes	Yes, if diet, oral antihyperglycemic agents, and insulin secretagogues are without results
Dietary recommendation	- Hypercaloric - Hyperproteic - Normoglucidic - Liposoluble vitamins	- Carbohydrate monitoring. - Normal balanced diet to allow the harmonious development of the child.	- Monitoring carbohydrates and calories. - Promotion of decreased weight
Physical activity	Yes		Yes
Macrovascular complications	No/Rare	Yes	Yes
Microvascular complications (retinopathy, nephropathy, and neuropathy)	Yes (less frequent and severe)	Yes	Yes
Causes of death	Pulmonary involvement	Cardiovascular disease; nephropathy	Cardiovascular disease

**Table 2:** The outcomes derived from diverse studies exploring potential methodologies for screening and diagnosing CFRD.

Method of Screening	Study	Year	Authors	Results	Ref.
OGTT	Insulin secretion abnormalities	2005	Tofé et al.	Hypoglycemia signifies a disruption in insulin secretion regulation.	[49]
OGTT	Early glucose abnormalities in cystic fibrosis are preceded by poor weight gain	2010	Hameed et al.	A peak blood glucose level < 8.2 mmol/L (147 mg/dL) is associated with a decline in the weight z-score and lung function over the preceding 12 months.	[50]
OGTT	Relation between 1-h plasma glucose during the OGTT and pulmonary function	2011	Brodsky et al.	Elevated blood glucose levels 1-h after the OGTT could serve as an early marker for the decline in respiratory function.	[7]
OGTT	AGT in children with CF and its relationship with the duration and severity of CF	2018	Banavath et al.	The majority of children with CF for >3 years and/or age >6 years developed AGT.	[48]
				AGT in children aged 6 to 9 years can identify those at a higher risk of developing CFRD.	
OGTT; FPG	Screening methods for the diagnosis of CFRD	2019	Granados et al.	The OGTT is primarily designed for detecting T2DM.	[2]
				The 2-h blood glucose level and fasting glucose level in the OGTT may not be as relevant in the context of CF as it is in T2DM.	
				Under these circumstances, an FPG of 7.0 mmol/L (126 mg/dL) or a postprandial blood glucose level of 11.1 mmol/L (200 mg/dL) may raise suspicion of CFRD.	
OGTT	Screening rate	2021	Racine et al.	A screening rate of 53%, with variations from 29.5% among children born before 1993 to 76.7% for those born after.	[41]
OGTT	Predictive value of 1-hour glucose elevations during the OGTT	2023	Lorenz et al.	The elevations in the 1-h glucose levels >140 mg/dL were linked to a progression toward CFRD in the subsequent 5 years.	[34]
				The 1-h glucose measurement distinguishes individuals at high risk from those at low risk of CFRD development.	
OGTT; HbA1c; CGM	Comparison between the OGTT and HbA1c	2018	Chan et al.	The limited agreement between the OGTT and HbA1c should not diminish the utility of HbA1c.	[8]
				HbA1c exhibited a notable correlation with multiple glucose measurements during CGM.	
FPG; HbA1c	Utility of different methods of diagnosis for CFR	2022	Sovtic	FPG concentrations can persist within the normal range for an extended duration in approximately half of CFRD patients.	[14]
				HbA1c exhibits a sensitivity of merely 50% and does not correlate adequately with the mean plasma glucose levels.	
				HbA1c—limited predictive value	
HbA1c	Sensitivity and specificity of HbA1c in CFRD	2016	Burgess et al.	Lower sensitivity (68.2%) and specificity (60.5%)	[51]
HbA1c; GA; OGTT	Investigation of alternate glycemic markers as screening tests for (CFRD)	2019	Tommerdahl et al.	The value of HbA1c 5.5% was found to differentiate patients with more stable lung functions from those with more impaired lung functions.	[39]
				GA exhibited a significant correlation with the 2-h OGTT values.	
HbA1c	The applicability of HbA1c as a method of diagnosis in children with CFRD	2019	Gilmour et al.	Limited data was available regarding the applicability of HbA1c between 5.5–6.4% for diagnosing CFRD in children.	[52]
FSF	Utility of FSF in screening for CFRD	2018	Lam et al.	In situations where the HbA1C measurements may be unreliable due to red blood cell turnover, FSF emerges as a reliable alternative for tracking the clinical outcomes.	[42]
GA	Utility of GA in the screening for CFRD	2021	Xiong et al.	GA should be considered an additional test rather than a substitute for HbA1c or OGTT.	[43]
HbA1c; OGTT; CGM	Correlations between HbA1c, OGTT, and CGM	2021	Gojsina et al.	The HbA1c levels were lower in the CGM–CFRD subgroup compared to the OGTT–CFRD subgroup.	[29]
CGM; OGTT	Comparison between the results of the OGTT and those obtained through CGM	2018	Leon et al.	CGM was effective in identifying glucose fluctuations that had gone unnoticed in the OGTT.	[53]
CGM; OGTT	The effectiveness of CGM in predicting the onset of CFRD	2022	Zorron et al.	The CGM is useful for identifying abnormalities in glucose metabolism that the OGTT might not detect.	[54]
				CGM offers the advantage of providing a more detailed characterization of glucose patterns.	
CGM	Utility of CGM for identifying carbohydrate metabolism abnormalities	2020	Prentice et al.	CGM effectively identifies carbohydrate metabolism abnormalities that are prevalent in children under 10 years of age.	[55]
CGM	Value of CGM in the diagnosis of CFRD	2023	Sebastian-Valles et al.	CGM represents the glycemic response as a continuous variable within a patient’s daily routine.	[56]
				The increased cost associated with CGM may restrict its routine use.	
CGM	Continuous glucose monitoring versus self-monitoring of blood glucose in the management of CFRD	2023	Vagg et al.	CGM may enhance glycemic control when compared to conventional finger-prick glucose monitoring.	[57]
CGM; OGTT	Comparative data on CGM versus the OGTT	2021	Elidottir et al.	CGM could serve as an additional parameter to the OGTT in the assessment of glucose abnormalities.	[9]
